# An Efficient Coding Hypothesis Links Sparsity and Selectivity of Neural Responses

**DOI:** 10.1371/journal.pone.0025506

**Published:** 2011-10-13

**Authors:** Florian Blättler, Richard H. R. Hahnloser

**Affiliations:** Institute of Neuroinformatics, University of Zurich/ETH Zurich, Zurich, Switzerland; Max Planck Institute for Human Cognitive and Brain Sciences, Germany

## Abstract

To what extent are sensory responses in the brain compatible with first-order principles? The efficient coding hypothesis projects that neurons use as few spikes as possible to faithfully represent natural stimuli. However, many sparsely firing neurons in higher brain areas seem to violate this hypothesis in that they respond more to familiar stimuli than to nonfamiliar stimuli. We reconcile this discrepancy by showing that efficient sensory responses give rise to stimulus selectivity that depends on the stimulus-independent firing threshold and the balance between excitatory and inhibitory inputs. We construct a cost function that enforces minimal firing rates in model neurons by linearly punishing suprathreshold synaptic currents. By contrast, subthreshold currents are punished quadratically, which allows us to optimally reconstruct sensory inputs from elicited responses. We train synaptic currents on many renditions of a particular bird's own song (BOS) and few renditions of conspecific birds' songs (CONs). During training, model neurons develop a response selectivity with complex dependence on the firing threshold. At low thresholds, they fire densely and prefer CON and the reverse BOS (REV) over BOS. However, at high thresholds or when hyperpolarized, they fire sparsely and prefer BOS over REV and over CON. Based on this selectivity reversal, our model suggests that preference for a highly familiar stimulus corresponds to a high-threshold or strong-inhibition regime of an efficient coding strategy. Our findings apply to songbird mirror neurons, and in general, they suggest that the brain may be endowed with simple mechanisms to rapidly change selectivity of neural responses to focus sensory processing on either familiar or nonfamiliar stimuli. In summary, we find support for the efficient coding hypothesis and provide new insights into the interplay between the sparsity and selectivity of neural responses.

## Introduction

Brains of higher vertebrates analyze the sensory world in hierarchical networks. In lower sensory brain areas, neurons tend to respond to generic stimulus features, whereas in higher areas they typically respond to only very small subsets of natural stimuli [1], [2]. Neural responses in lower areas are usually characterized by the stimulus property that correlates most strongly with spike responses, based on which neurons are termed feature detectors for that particular property. For neurons in higher brain areas, the concept of feature detector is often abandoned in favor of stimulus selectivity (assessed in terms of the stimulus that elicits the maximal response), e.g., neurons in higher auditory areas are selective for a particular birdsong [3], or, in visual areas, they are selective to the face of a particular person [4].

Feature tuning in sensory neurons can be explained by a neuronal strategy to efficiently encode natural stimulus ensembles, such as e.g. in simple cells of primary visual cortex or in auditory cells of the cochlear ganglion [5], [6]. However, it is unclear whether (unsupervised) efficient coding principles can also explain complex response selectivity, as proposed in [7], in particular when selectivity applies to a behaviorally relevant stimulus such as the tutor song for a juvenile songbird. Naively, the selectivity for tutor song (TUT) or the bird's own song (BOS) that is commonly observed in song-control brain areas [8], [9] seems to violate the efficient coding hypothesis, according to which responses to frequent stimuli should be minimized [7], [10], not maximized. Hence, it is an open question how complex selectivity (maximal response to BOS) can be reconciled with the sparse firing that often accompanies such selectivity.

The rationale of our work is that the firing sparsity of cells is governed by slow developmental mechanisms that construct efficient representations of natural stimulus statistics and by faster mechanisms influenced by the recent stimulus history and the state of the animal and its environment. In many cells, excitatory and inhibitory currents are balanced in proximity of the firing threshold, though currents can be dominated by inhibition, as for example in some sparsely firing neurons [11]. Also, shifts in the excitatory/inhibitory balance are commonly observed, for example as a function of stimulus intensity [12] or during sensory adaptation [13]. Mechanistically, the excitation-inhibition balance can be controlled by neuromodulatory mechanisms such as the serotonergic system [14], and, a link has been suggested to exist between the excitation-inhibition balance and attention [15]. We study the dependence of response selectivity in model neurons on fast shifts in the balance between excitatory and inhibitory inputs.

In our model, we reflect the asymmetry imposed by the firing threshold (supra- versus sub-threshold responses) by an asymmetric cost on sub- and suprathreshold synaptic currents. We train neurons using a particular firing threshold and thereafter we explore the consequences of shifts in the excitatory/inhibitory balance by evaluating neural responses for an entire range of firing thresholds.

**Figure 1:**
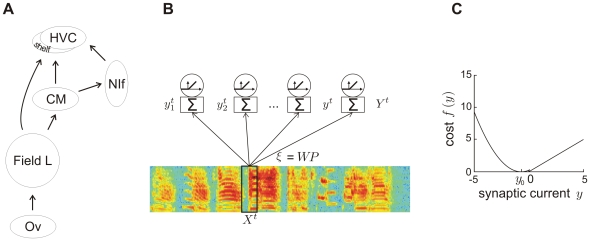
Efficient coding of cochlear spectrograms. (A) Schematic of the auditory pathway in the songbird forebrain. Auditory input to the pallial field L is provided by the thalamic nucleus ovoidalis (Ov). From field L, auditory information is relayed to the caudal mesopallium (CM), and from there to the nucleus interface of the nidopallium (NIf) and to HVC. (B) Network model. At time 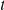, the auditory input to the network is a 50-ms window 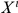 of the sound spectrogram. This input is multiplied by synaptic weights 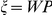 to result in total synaptic currents 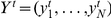 onto 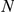 neurons. 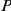 stands for whitening and dimensionality reduction (principal component analysis), and 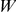 stands for a sparseness transformation. Neural firing rates are given by rectified synaptic currents. (C) Cost function for a threshold 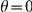. Subthreshold synaptic currents 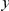 are punished quadratically and suprathreshold currents are punished linearly. The parameter 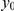 defining the subthreshold current eliciting minimal cost is set to the expected subthreshold current 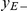. Synaptic currents are reported in units of mean-subtracted standard deviations (z-scores). A threshold 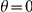 implies that suprathreshold currents are depolarizing (positive), whereas subthreshold currents are hyperpolarizing (negative). 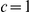.

Our model is applicable to sensory systems in general but presented as a model of the auditory forebrain pathway of songbirds. This pathway extends over the nucleus ovoidalis to field L, and from there up to HVC [9], [16], [17], Figure 1A. A crucial function of this pathway is to subserve song learning, a process in which birds first memorize a template of a tutor's song (TUT) and then refine their vocalizations to gradually approximate the template [18]. During these two learning phases, neurons in the lower auditory brain area field L develop a broad response selectivity for natural sounds, in particular the songs of conspecific birds (CON) [19]. By contrast, neurons in higher areas develop selectivity for the TUT and the BOS in particular [20], [21]. Preference for BOS over CON and REV is first observed in the caudal mesopallium (CM). BOS preference is even stronger in the nucleus interface of the nidopallium (NIf) and highest in the premotor area HVC [8], [9]. Notable about this gradual increase in BOS selectivity along the main auditory pathway is a conjunctive increase in firing sparsity, illustrated by NIf neurons firing rather densely and HVC projection neurons bursting only once during a song or song motif [8], [22].

## Results

We model the auditory pathway as a feedforward network that receives (indirect) auditory input from the cochlea in the form of spectro-temporal sound patterns. These patterns are mean-subtracted, multiplied by synaptic weights, and summed, to result in the total synaptic current impinging onto neurons. Given this linear model we interpret the set of synaptic weights onto a neuron as its spectro-temporal receptive field (STRF). We devised an algorithm that optimizes synaptic weights for their propensity to decorrelate (whiten) and sparsify synaptic inputs: First, we whitened cochlear inputs using a projection matrix 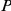 (principal component analysis, PCA), and then we applied a sparseness transformation using a matrix 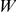 that minimizes an asymmetric cost imposed on total synaptic currents, Figure 1B. This cost depends on the firing threshold 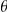. Subthreshold synaptic currents are punished quadratically, whereas suprathreshold currents are punished linearly. The location of minimal quadratic cost is freely selectable using the parameter 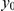, Figure 1C. For simplicity, in most simulations we neglected this parameter by setting 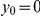 (we set 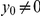 when our goal was to optimally reconstruct cochlear inputs from suprathreshold currents, in which case there is a better choice for 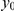 than zero, see Methods).

This model guarantees zero mean synaptic current and minimal variance (see Equation 8). Intuitively, to minimize the cost, frequent cochlear input patterns must elicit weak subthreshold currents (quadratic cost is smaller than linear cost). By contrast, rare cochlear input patterns must elicit strong suprathreshold currents (linear cost smaller than quadratic). We defined firing rates to be equal to suprathreshold synaptic currents, meaning that the linear cost of suprathreshold currents is in effect a cost on the average population firing rate. In the Methods we show that the quadratic cost of subthreshold currents is a cost on a reconstruction error associated with simple decoding of cochlear inputs from firing rates. Thus, the algorithm tries to maximally sparsify population responses without discarding any relevant sensory information. We minimized the cost function over training data consisting mostly of renditions of a particular zebra finch song (BOS) and a few CONs. After training, we evaluated neural responses for a wide range of firing thresholds (thresholds during training could deviate from thresholds during evaluation). Changes in firing thresholds modeled changes in the excitatory/inhibitory balance. Details are presented in the Methods.

### Spectro-temporal receptive fields (STRFs)

**Figure 2:**
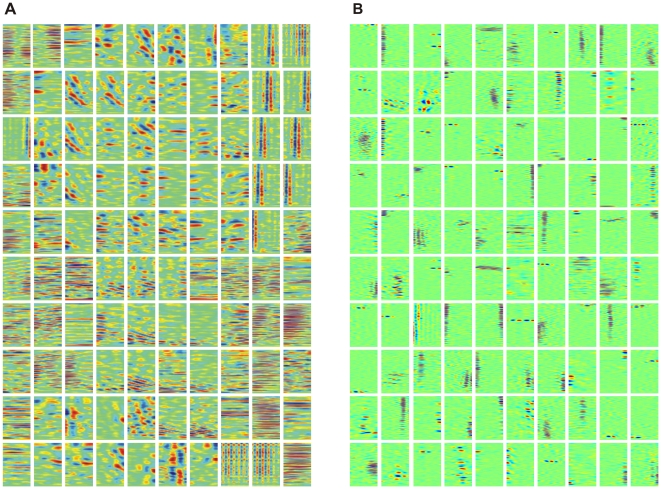
Spectral temporal receptive fields. (A)Spectral temporal receptive fields (STRFs) of 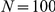 neurons, arranged by nearest-neighbor similarity (circular boundary conditions). Neurons tend to be either temporally tuned (vertical stripes, top right), spectrally tuned (horizontal stripes, middle rows), or display more complex spectro-temporal patterns. Spectral resolution is 172 Hz, offset between subsequent cochlear inputs is 1.5 ms. (B) STRFs obtained with a linear cost on synaptic weight magnitudes. The linear cost forces many synaptic weights to be close to zero (green), leading to low-density STRFs most of which contain a smaller number of excitatory and inhibitory subfields than in A. Interestingly, excitatory and inhibitory subfields tend to be close to each other and aligned horizontally or vertically, similar to observations in field L neurons. The 100 presented STRFs were randomly chosen out of the total 800. 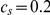.

After training, neurons displayed a large diversity of STRFs. Typically, STRFs were patchy and had multiple adjacent inhibitory and excitatory spectral/temporal subfields. In many neurons, STRFs were regularly arranged into horizontal or vertical stripes (Figure 2A), similar to receptive fields in field-L neurons that encode elementary spectro-temporal sound features such as sound onsets or a particular sound pitch [23]–[26].

Model STRFs had excitatory and inhibitory subfields that together covered the entire spectro-temporal window of the STRF. Typically STRFs in field L are of considerably lower density in that they mostly possess only two or three subfields instead of more than six. We therefore explored whether STRFs in our model would be of lower density when we added a third term to the cost function, a term corresponding to a linear cost on absolute synaptic weights. We found indeed low-density STRFs if the parameter 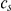 weighing this third term exceeded roughly 0.1, Figure 2B. Density of STRFs could be controlled independently of the sparseness of model responses, explored next.

**Figure 3:**
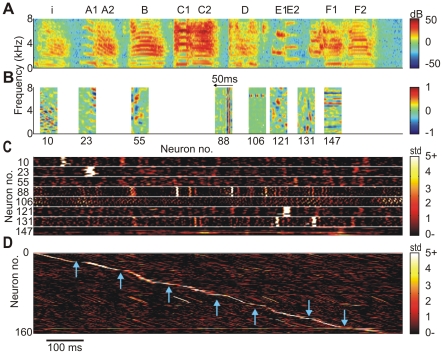
Receptive fields and neurogram. (A) Power spectrogram of a bird's own song (BOS). (B) STRFs 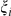 of eight representative neurons (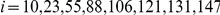). The horizontal alignment of STRFs with the spectrogram in A is such that the trailing edges of the STRFs correspond to the respective peak times of synaptic currents. The temporal axis of the STRFs is inverted for better comparison with the BOS spectrogram. (C) Stack plot of synaptic currents of representative neurons in B in response to ten different versions of BOS, vertically aligned to A. (D) Neurogram of synaptic currents in response to the BOS in A. The 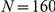 neurons are sorted according to the peak times of their synaptic currents. Fewer neurons display synaptic current peaks during syllable gaps (blue arrows) than during syllables.

We explored the correspondence between STRFs and the stimulus features to which neurons responded most. In most cells, presentation of different BOS versions elicited reliable responses to specific song notes, Figure 3A–C. For example, the total synaptic current in neuron 10 with a checkerboard-like STRF reliably peaked after the down sweep of the introductory note and to a lesser extent it also peaked at the offsets of some other syllables. Neuron 23 with a narrow and slanted STRF responded most strongly to the down-sweep of the harmonic stack in Syllable A1. The STRF of Neuron 88 had sharp vertical subfields, the synaptic current to this neuron peaked during rapid increases of sound intensity such as during the onsets of Syllables C and D. Another neuron with a vertically dominated STRF (Neuron 131) responded a few milliseconds after the onsets of Syllables E and F. This neuron was able to respond to different syllable onsets than Neuron 88 by virtue of its sensitivity to a low-frequency tone immediately followed by a high-frequency tone, which is a common characteristic of both Syllables E and F. Very particular was Neuron 106. Its receptive field and that of several other neurons were centered on a single frequency band close to 7 kHz. It turned out that this cell responded to electrical noise by which our recordings were affected; during BOS presentation, the total synaptic current to this cell was small and increased mainly during syllable gaps where no signal except the noise was present. The structure of the sparsely checkered STRF of Neuron 121 was particularly well adapted to Syllable E, the neuron responded almost exclusively during the transition between sub-Syllables E1 and E2. Finally, Neurons 55 and 147 did not show either robust or strong responses to BOS; a more thorough analysis revealed that they responded strongly to a CON in the training set. When we ordered all neurons by the time at which their synaptic currents peaked in response to a particular version of BOS, we found that the resulting stack plot exhibited a staircase-like shape: peak currents were widely distributed across cells with many more peaks seen during syllables than during syllable gaps, Figure 3D.

**Figure 4:**
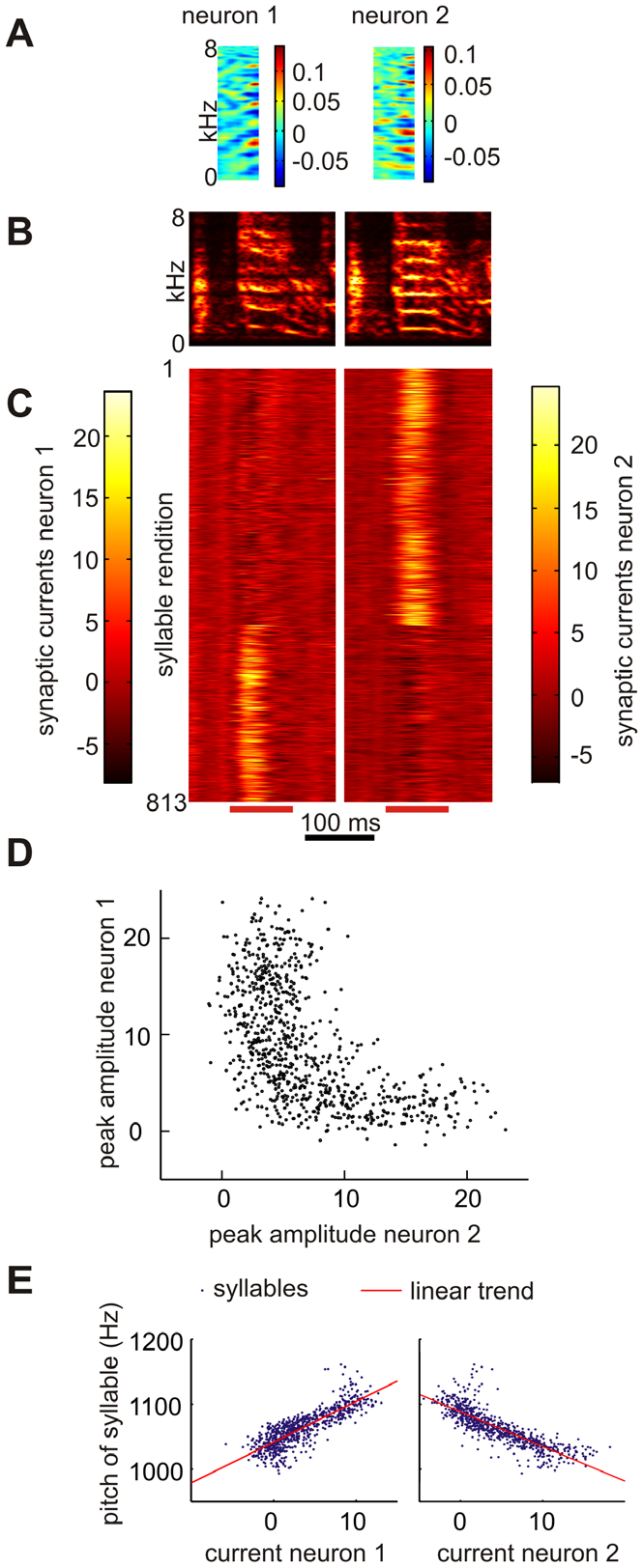
Neurons encode behavioral variability, for example song pitch. (A) Two receptive fields formed by training a network on all songs produced by a bird on a single day. (B) Spectrograms of a song syllable containing a harmonic stack. The left version has median pitch 1024 Hz, the right version 1138 Hz. (C) Stack plot of synaptic currents in the two neurons elicited by 813 syllable renditions. The stack plots have been sorted identically to reveal that for a given syllable rendition either the left or right neuron exhibits a peak in synaptic current, but not both. Peaks in synaptic currents are computed in intervals indicated by red bars on the bottom. (D) Scatter plot of peak synaptic currents in the two neurons. The distribution is sparse (‘L’-shaped). (E) Median synaptic current in same intervals versus median song pitch of the harmonic stack. The two neurons are detectors of low and high pitch versions of the stack, respectively. Red and blue lines are linear regressions (Neuron 1: 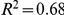, 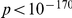, Neuron 2: 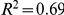, 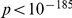), 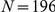, 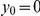.

Some neurons were not just tuned to a particular syllable within the motif, but had even more specific tuning to particular subsets of that syllable. Finches often produce harmonic stack syllables and can subtly vary the pitch of these syllables in a well-controlled and goal-directed manner [27], [28]. When we trained a network of 196 neurons on the songs of a bird produced during an entire day, we found that synaptic currents of two neurons peaked during a harmonic-stack syllable produced by that bird (neurons with such harmonic-stack receptive fields have been illustrated in [26]). Interestingly, for any given stack syllable, the synaptic current in only one of the neurons peaked, but not in both, Figure 4. The two neurons divided the representation of that syllable between each other, one represented the high-pitch version of the stack, the other the low-pitch versions, Figure 4E. Hence, our algorithm is able to ‘allocate’ more than a single neuron to a song feature, depending on the extent of its variability.

**Figure 5:**
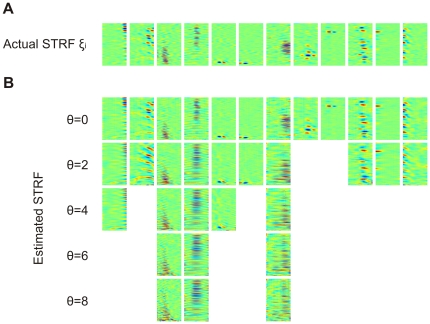
Actual STRFs and STRFs estimated using reverse correlation. (A) A selection of twelve STRFs 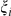 obtained after convergence of the algorithm (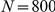). (B) Estimated STRFs (reverse correlation) based on the predicted firing rates 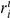. Shown are only estimated STRFs for neurons associated with a correlation coefficient 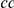 between predicted and actual firing rates of 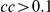. 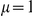. 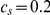.

STRFs are optimal models of the linear part of neural responses. Can we recover the STRFs found by our algorithm in simulated neural responses which contain a threshold nonlinearity? To this end, we estimated STRFs from nonlinear responses to birdsong stimuli for a range of firing thresholds 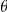. We estimated STRFs using reverse correlation. Mostly, we found strong resemblance between estimated and actual STRFs, Figure 5. Strong resemblance was seen in all cases in which the correlation coefficient 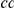 between estimated and actual responses was above 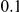, i.e. in cases in which the linear model was reasonably good. Moreover, estimated STRFs did not change much with increasing firing threshold 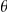, except that with increasing 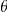 the STRFs had a small tendency to extend over larger time-frequency regions (Figure 5B) than the original low-density STRFs (Figure 5A). We found similarly satisfying results when estimating high-density STRFs (not shown). Hence, estimated STRFs were quite insensitive to the nonlinearity and to changes in firing threshold.

### Distribution of synaptic currents

**Figure 6 pone-0025506-g006:**
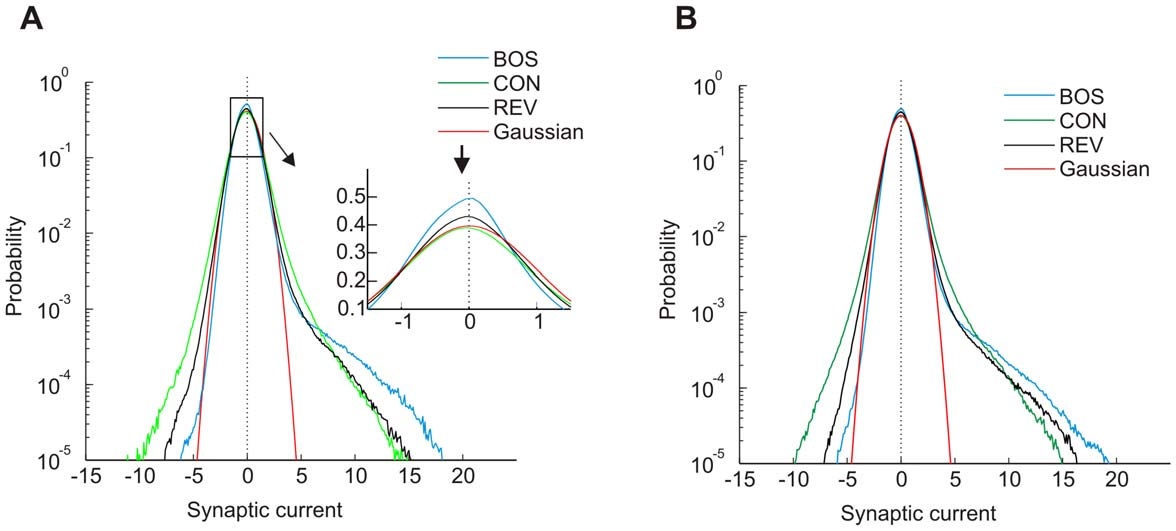
Probability density of total synaptic currents. (A) The probability density of total synaptic currents 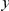 averaged over all neurons has a heavy tail on the positive side. Shown are the densities for BOS (blue), CON (green), and REV (black). Near zero synaptic currents, the curves are approximatively unit Gaussian (red), though their excessive peaks are slightly shifted to the negative side (inset, arrow). The curves cross each other such that large positive synaptic currents are preferentially elicited by the BOS and small positive currents by REV and CON. 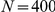, 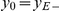. (B) The distributions of synaptic currents for sparse STRFs (Figure 2B) are qualitatively similar to (A). The only noticeable difference is that the distribution for REV is closer to BOS, reflecting a lower selectivity for temporal order. 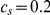.

The distribution of total synaptic currents over all neurons and over all training stimuli was highly asymmetric and contained many positive but few negative outliers, Figure 6A. The distribution of BOS-evoked currents could be reasonably well approximated by a unit Gaussian on the negative side and a long-tail exponential on the positive side. This combined Gaussian-exponential behavior follows from the fact that minimization of the quadratic-linear cost function is equivalent to locally maximizing the log-likelihood of a Gaussian model density below the threshold and of an exponential model density above the threshold, under the global restriction of zero mean and fixed variance. Interestingly, large synaptic currents were mostly elicited by the BOS rather than by other stimuli, illustrating that neurons were best tuned to the features of the most prominent stimulus in the training set, which was the BOS. The same finding was true for low-density STRFs (when 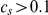 instead of zero): BOS-elicited currents exhibited a heavier positive tail than currents elicited by CON and REV (Figure 6B). Hence, model responses were robustly tuned for the BOS.

We also explored the influence of other model parameters such as 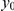, which sets the location of minimal quadratic cost. When we changed 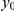 to nonzero values different from the firing threshold 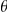 during training, we found that BOS tuning of synaptic currents was qualitatively unchanged. The only effect of changing 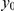 was to slightly increase the distribution of synaptic currents around 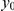 (where cost is minimal) and to slightly decrease it around 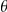 (not shown).

### Distribution of firing rates

**Figure 7 pone-0025506-g007:**
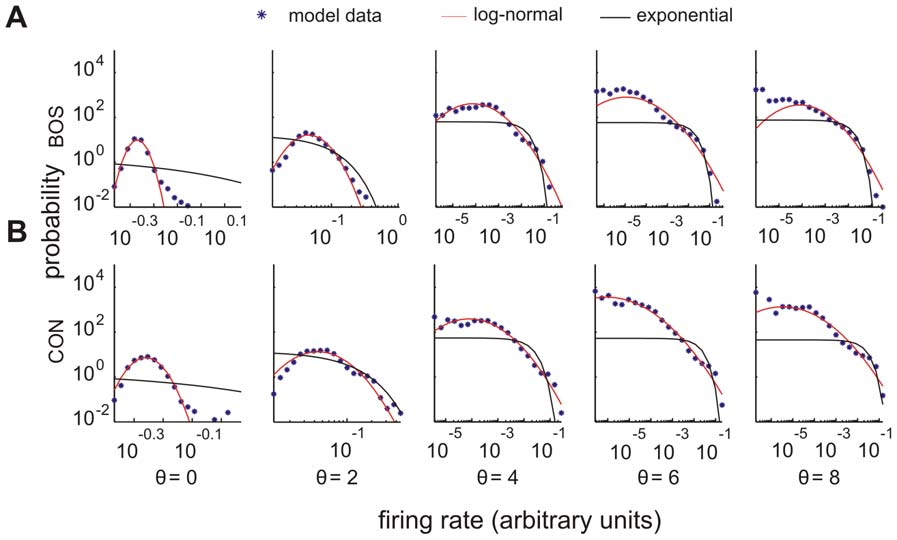
Probability densities of mean firing rates. Mean firing rates in response to (A) a BOS stimulus and (B) a CON stimulus. For each cell we computed the mean firing rate to one stimulus trial. Our simulation data (blue asterisks) are better fit by log-normal densities (red) than by exponential densities (black). Firing rates are plotted in arbitrary units. Fit parameters for log-normal densities were determined by the mean and variance of logarithmic firing rates, and for exponential densities they were determined by the mean firing rates. Thresholds varied from 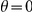 to 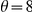. Noise amplitude 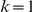.

We computed firing rates in model neurons by thresholding total synaptic currents. A recent analysis of sparsely firing cells in primary auditory cortex of unanesthetized rats has revealed that both background and stimulus-evoked firing rates are well fit by log-normal distributions [29]. We speculated that log-normal firing may be a corollary of efficient coding that could be reproduced in simulations. We inspected the distributions of firing rates across all neurons for all BOS and CON stimuli. Indeed, we found that the density of firing rates was best fit by a log-normal distribution, which was especially true for low firing thresholds, Figure 7. Note that recently published firing rate distributions of field L neurons in zebra finch were fit by Gamma distributions [30]. However, the published data suggests that a fit with a log-normal distribution should be equally good.

### Independence of neural responses

**Figure 8 pone-0025506-g008:**
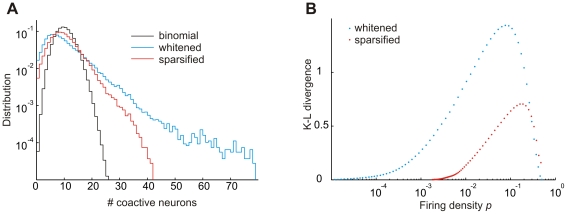
Sparsification reduces firing dependences. (A) The sparseness transformation renders the size distribution of coactive neuron groups (whitening+sparseness) closer to binomial. The probability 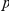 of the binomial distribution (that a neuron is active per unit time) was estimated in terms of the firing density (the fraction of suprathreshold events over all neurons and training stimuli). Probabilities 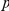 were nearly identical for whitening and whitening+sparseness when 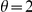 (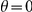 and 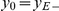 during learning). (B) The Kullback–Leibler divergence between size distributions is smaller when comparing the whitening+sparseness model to the binomial model than when comparing the whitening model to the binomial model, for nearly all firing densities tested.

Training the network increased the independence of neural responses. If responses were perfectly independent among neurons, the size distribution of coactive neurons would be binomial (the size distribution is the probability that a given number of neurons fire synchronously). The sole parameter of this binomial model is the single-neuron firing density that we estimated in terms of the fraction of suprathreshold events observed in the entire neuron population and for all training stimuli. In comparison to this binomial model, the observed size distribution elicited by whitened cochlear inputs was substantially wider, illustrating strong firing dependencies. The sparseness transformation significantly narrowed the observed size distribution towards the binomial case, Figure 8A. This increase of independence (decrease in Kullback-Leibler divergence to the binomial model) was true for both high and low firing densities, and true for nearly all firing thresholds tested, Figure 8B, revealing that the sparseness transformation is a robust mechanism to increase independence of neural responses. Qualitatively, this behavior of the network to render responses more independent applied to all firing thresholds used during training (we tested thresholds up to 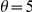).

### Decoded firing and reconstructed spectrograms

**Figure 9 pone-0025506-g009:**
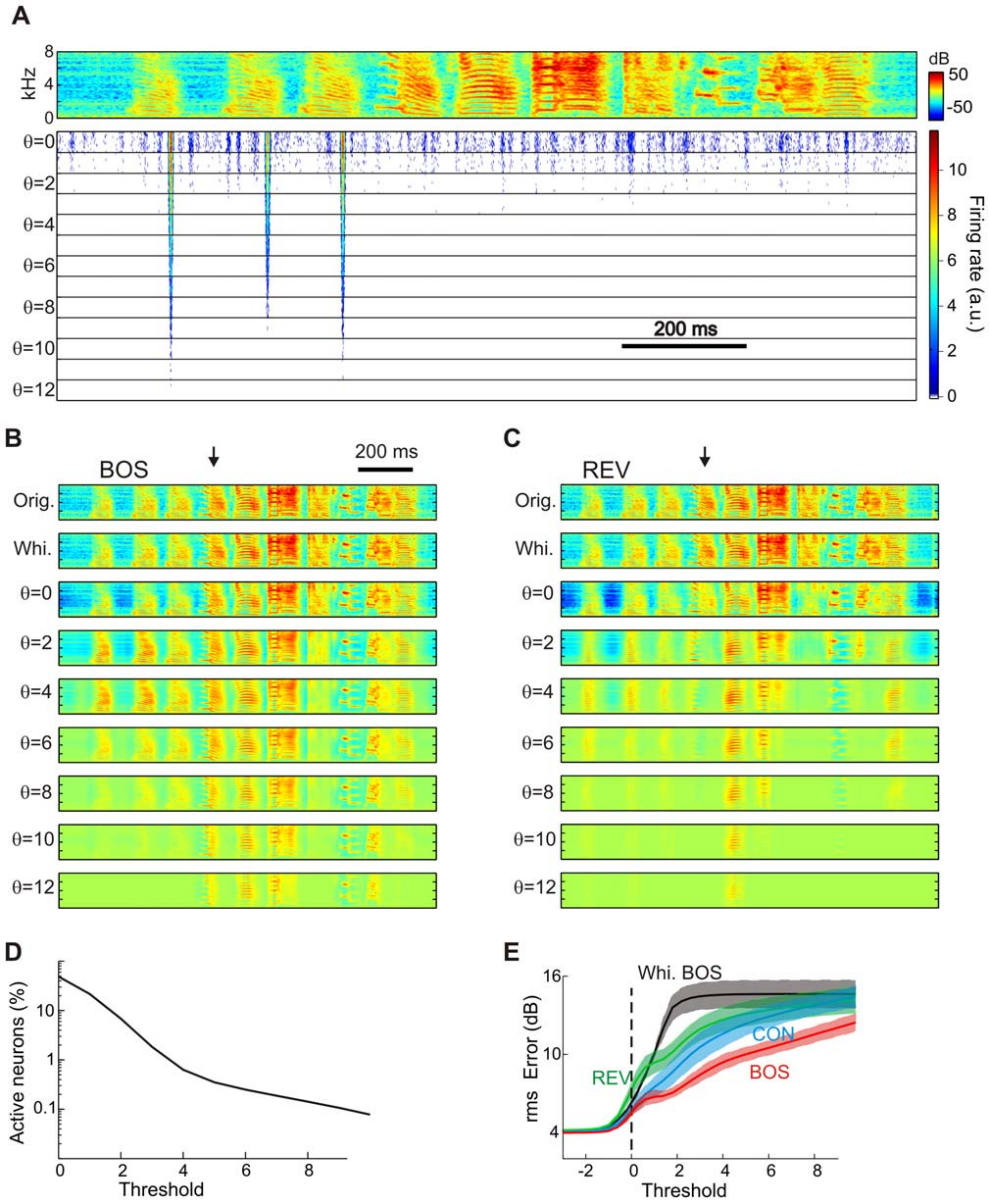
Reconstructing the cochlear spectrograms from firing rates. (A) Firing-rate of one example neuron in response to BOS for increasing firing thresholds (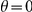 to 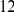). The BOS spectrogram is shown on top. This neuron is tuned to a feature present in introductory notes and responds to it up to thresholds higher than seven. For each threshold, ten different responses are plotted, corresponding to ten different instantiations of synaptic noise. 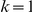. (B) The reconstruction of a BOS spectrogram (orig., top) using all neurons, based on a firing threshold of minus infinity (whi., 2nd from top) is fairly complete with little information loss (arising from dimensionality reduction). With increasing thresholds (below), more and more syllables are lost in the reconstruction, but the reconstructed spectro-temporal patterns remain clearly recognizable. The arrow points to a down-sweep syllable. (C) Reconstructions of REV (flipped horizontally for comparison with B) are worse than reconstructions of BOS at the same threshold; for example the down-sweep syllable is not well reconstructed (arrow), presumably because zebra finches produce almost no up-sweeps. (D) The fraction of active neurons (averaged over all BOS stimuli) decreases with increasing threshold such that at 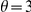 about 1% of neurons are active on average. This fraction decreases to 0.1% at about 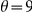. (E) The reconstruction errors averaged over different stimulus ensembles are monotonic functions of the firing threshold. For a given positive threshold, reconstruction errors increase from BOS to CON to REV. 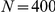 threshold-linear neurons.

Our network allowed us to decode firing rates and reconstruct the spectro-temporal sound patterns that elicited them, using the pseudo-inverse of the sparseness transformation 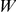 (see Methods). We evaluated the reconstructions for various firing thresholds (after training at a fixed threshold of zero), Figure 9A-C. For a threshold of zero, neurons produced dense firing patterns in response to BOS, with roughly 50 percent of neurons active at any time, Figure 9D. The percent active neurons decreased from 20% for 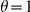, to 1–2% for 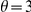, and down to 0.4% for 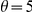. At thresholds higher than roughly three, reconstruction errors associated with non-BOS stimuli were often due to missed syllables because none of the neurons fired in response to these syllables.

In all cases, reconstruction errors increased with increasing firing threshold in a monotonic manner, Figure 9E. For a given threshold, reconstructions from sparseness-transformed cochlear inputs were much better than reconstructions from merely whitened inputs. This superiority was true even though for thresholds up to approximatively 1.3, mean firing rates were lower for sparseness-transformed inputs than for merely whitened inputs.

Moreover, for a given threshold, reconstruction errors tended to be larger for the BOS played back in reverse (REV) than for BOS or CON, illustrating that reconstructions were optimized for stimulus ensembles experienced during training but not for novel ensembles. In the Methods we show that the reconstruction error is approximatively equal to a term that grows not only with the number of subthreshold events, but also with their variance; hence, stimuli that induce narrow subthreshold distributions (such as the BOS, Figure 6) lead to smaller reconstruction errors.

### Smart noise suppression by selective neuron exclusion

**Figure 10 pone-0025506-g010:**
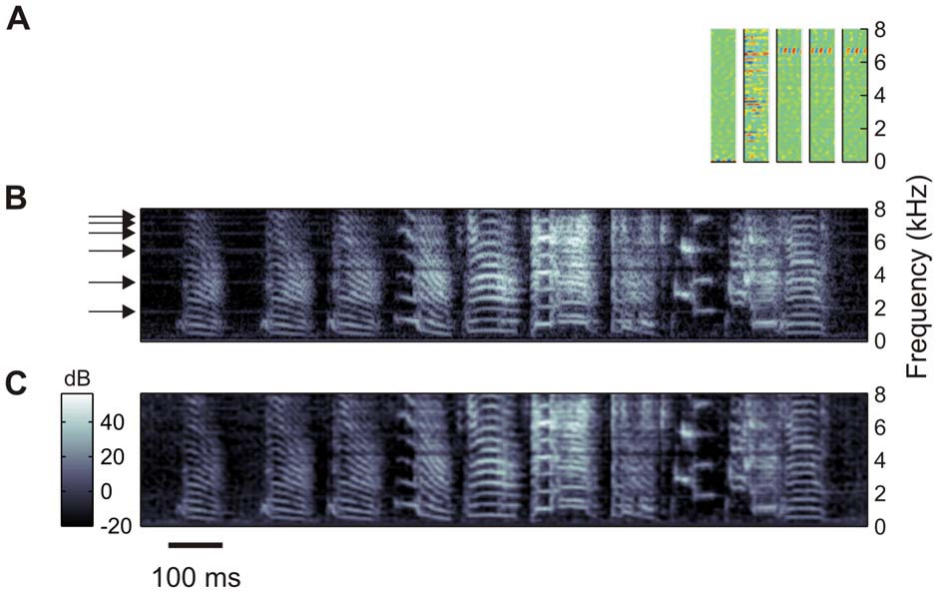
Smart suppression of electrical noise affecting the recordings. (A) The STRFs of five neurons that encoded monitor noise. (B) Original BOS spectrogram. The noise is manifest as gray horizontal bands (black arrows) during syllable gaps. (C) Reconstruction of the BOS (Equation 14) from elicited responses in the network. The thresholds of all neurons were set to 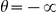, with exception of the five neurons in A in which the thresholds were set to 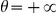. The monitor noise has vanished in the reconstructions, without affecting the birdsong signal.

As illustrated in Figure 10, five STRFs were encoding electrical noise elicited by the computer monitor. When we omitted these five neurons for BOS reconstructions, we were able to effectively suppress the monitor noise. To demonstrate the effectiveness of this smart noise suppression, we iteratively estimated the sound waveform associated with the reconstructed BOS [31]. The original and the noise-suppressed waveforms are available as supporting information files Audio S1 and S2 in wav format.

### Song selectivity is linked to firing sparseness

We explored the response selectivity of model neurons using the psychophysical 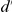 measure [32] that is routinely applied in birdsong studies. According to this measure, the selectivity for a stimulus over another is given by the difference in mean firing rates elicited by these stimuli, normalized by their standard deviations (see Methods). We assessed the selectivity of neurons to BOS versus matched spectro-temporal stimuli such as CON and REV. Variability of responses to a fixed stimulus was generated by a white-noise current source (see Methods).

**Figure 11 pone-0025506-g011:**
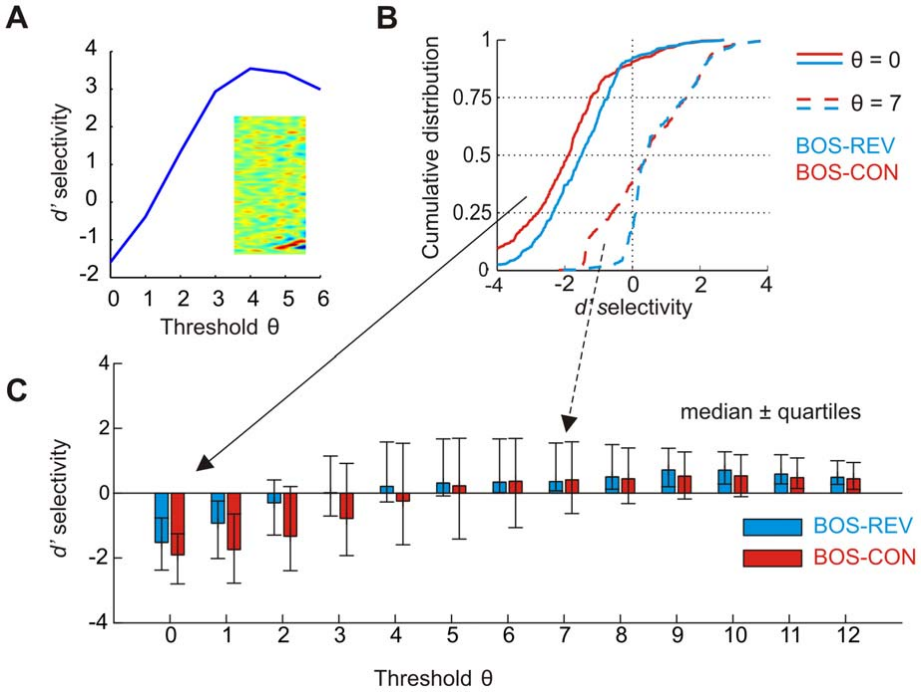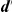 selectivity for BOS reverses at high firing thresholds. (A) Example model neuron with reversing BOS-CON selectivity. This neuron's STRF (inlay) codes for an up-sweep from 500 to 800 Hz over 20 ms. The resulting BOS-CON 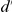 selectivity is negative for low thresholds 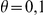 and turns positive for thresholds 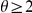. (B) Example cumulative distributions of BOS-REV (blue) and BOS-CON (red) 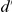 selectivities across 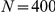 neurons for 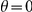 (solid lines) and 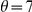 (dashed lines). For 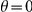 the selectivities are biased towards negative values, whereas for 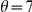 the distributions are biased towards positive values. (C) Bar plot summarizing BOS-REV (blue) and BOS-CON (red) selectivities for a wide range of firing thresholds. The colored bars indicate the median 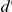 selectivity and the error bars delimit the first and third quartiles. Selectivity reverses at around 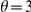 (REV) and 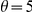 (CON).

We found a wide range of selectivity behaviors. Many neurons responded more strongly to CON than to BOS, but this CON preference often reversed to BOS preference at high firing thresholds, Figure 11B. For a firing threshold of zero, the median 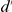 selectivity for the BOS was negative across the population, both with respect to REV and to CON, Figure 11B. Hence, at this low threshold, the majority of neurons preferred REV and CON over BOS. BOS anti-preference remained true for a range of firing thresholds 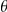 up to three. However, all of the median and mean BOS-REV and BOS-CON selectivities became positive at thresholds 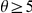, Figure 11C. Thus, the response selectivity of the network was non trivial in that it reversed at higher thresholds. Overall, BOS preference at high firing thresholds was seen both for analog models (firing rates are defined as thresholded synaptic currents) and for binary models (firing rates are defined as binarized synaptic currents). From the point of view of stimulus selectivity, the low-threshold regime of our network is a model of densely firing field-L neurons and the high-threshold regime is a model of sparsely firing HVC neurons.

Our training set contained many versions of two different CONs. For BOS-CON selectivity reversal it did not matter whether selectivity was tested on three novel CONS as in Figure 11, or on twelve novel CONs, or on the two trained CONs, because for all these cases the median and mean BOS selectivities reversed at around 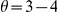. However, when CONs from many more birds (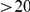) were in the training set, then the median BOS-CON selectivity became positive only at very high thresholds (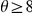 for 22 CONs), whereas the mean selectivity became positive already at 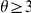. Thus, when responses to many different songs are sparsified, then increasing numbers of neurons develop a feature preference that best matches a CON in the training set rather than the BOS; however, this match is not particularly good as illustrated by BOS that is preferred on average already at relatively low thresholds.

For any given threshold 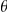, the median 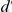 selectivity (be it positive or negative) depends on the noise level. When increasing the noise level, the median 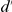 selectivity goes toward zero, and, when decreasing the noise level, the median 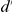 selectivity diverges from zero. 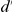 magnitudes are also influenced by the number of different song renditions used to probe selectivity. When selectivity is probed with a single BOS and a single CON file and noise is small, 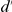 values can become arbitrarily large. Hence, our model allows for close to arbitrary scaling of 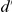 values by manipulating the intrinsic noise.

We tested the dependence of BOS selectivity on the temporal summation window and found that our results did not depend critically on STRF width. Model STRFs were 50 ms wide. For 100-ms wide STRFs, BOS-CON and BOS-REV selectivities reversed at around 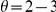; and, for 25-ms wide STRFs, selectivity reversal was seen at around 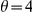. Hence, with increasing temporal summation window, selectivity reversal was seen at lower thresholds. Note that 25-ms STRFs are much shorter than estimated integration times in BOS-selective neurons [23], [33].

We also tested the effect of neuron number on 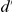 selectivity. Doubling that number from 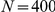 to 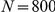, or reducing it to 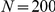 or 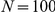 preserved selectivity reversal in the range 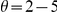, for all CON ensembles tested and for both firing-rate models. Also, we found that the value of the threshold 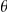 during learning has little influence on selectivity reversal after learning. For 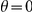, 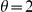, and 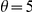 during learning, selectivity for BOS reversed to positive values at around 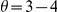 after learning. In summary, selectivity reversal at high thresholds was very robust and did not depend on model details.

**Figure 12 pone-0025506-g012:**
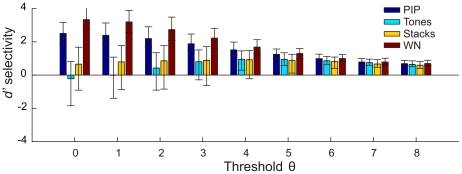
Selectivity for CON vs. different artificial stimuli. Depicted are median selectivities 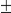 quartiles. PIP = tone-pip stimuli, WN = white noise.

We assessed whether our model neurons preferred CON over artificial stimuli, as has been reported in field L [34], [35]. We found high median selectivity for CON versus tone pips, tone stacks (ripples), and white noise (Figure 12). This CON preference was true for all thresholds 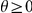 examined. Solely tones (sparse colored noise) were preferred over CON for thresholds up to 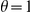. For higher thresholds 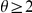, CON-tones selectivity reversed and CON was strongly preferred.

### Comparison with an ICA algorithm

The key element in our model seemed to be the positive tail of synaptic currents. Because this tail has a non-Gaussian shape, our model can be seen as part of a broader class of independent-component analysis (ICA) algorithms that extract maximally non-Gaussian components from data [36]. To test whether BOS preference at high thresholds arises also in other ICA algorithms, we trained an identical network using the classical ICA algorithm by Bell and Sejnowski (results not shown) [37]. In this algorithm, as in most similar algorithms, the final distribution of synaptic currents is symmetric, with heavy tails on both sides. For this reason we applied the firing threshold to absolute synaptic currents (see Methods). We computed BOS selectivities for different firing thresholds 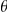 and found that BOS-CON and BOS-REV selectivities reversed as in our model, but at higher thresholds: The median and mean BOS-REV selectivities reversed at 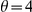, whereas the mean and median BOS-CON selectivities reversed at around 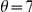. Thus, the emergence of BOS preference in the ultrasparse regime of simple networks did not depend on how efficient coding was enforced, but appears to represent a generic consequence of non-Gaussian statistics and the choice of the training set.

### Simulation of a two-layer network

**Figure 13:**
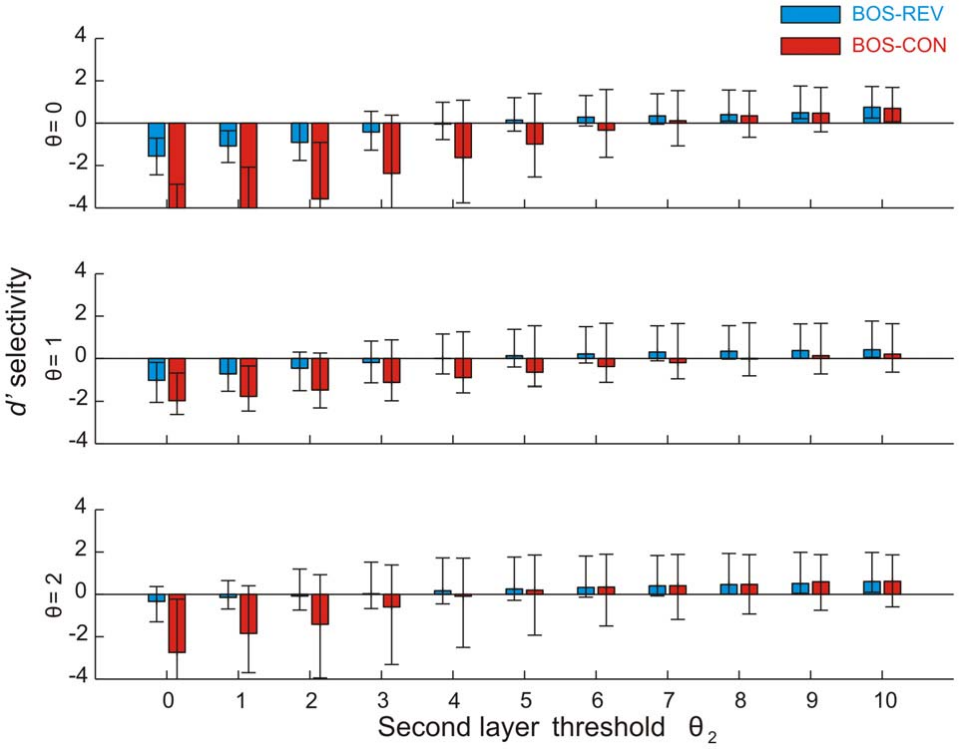
Selectivity in two layers. Median and quartile selectivities in a network of two layers for various firing thresholds 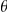 in the first layer and 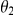 in the second layer. In each simulation, second-layer responses were evaluated using the first-layer threshold applied during training. As can be seen, BOS preference in the second layer is restricted to the high-sparseness regime there (right part of the three subplots).

To account for the layered architecture of the auditory pathway, we also explored a two-layer network, in which the second layer was trained on thresholded first-layer outputs. In simulations, first-layer outputs were first summed over consecutive time bins (to extend receptive field widths in the second layer) and were then subjected to whitening and sparseness transformations (as we did for the first layer, see Figure 13). In simulated networks in which the first layer was a universal encoder (small 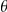), we found that response selectivity in the second layer reversed at high firing thresholds in preference of BOS. Hence, our high-threshold model of response selectivity in HVC can also be expressed in an architecture that is consistent with the feedforward organization of the auditory pathway.

## Discussion

Our model offers an understanding of auditory response selectivity based on an efficient coding hypothesis. We briefly outline the computational roots of the network we described, compare neural responses to those of songbird auditory neurons, and discuss possible consequences of efficient synaptic currents for sensory and motor processing.

### ICA algorithms

Our model falls into the broad category of ICA and sparse coding algorithms which try to linearly transform given (sensory) inputs into independent outputs (e.g., synaptic currents). In most ICA algorithms, independence of outputs is enforced by a symmetric cost such as kurtosis or entropy [37]–[39]. Because of this symmetry, most ICA algorithms fail to account for the asymmetry imposed by the spike threshold. By contrast, our algorithm explicitly includes a rectification nonlinearity which truncates as little information as possible because we minimize an approximate error of reconstructed spectrograms. Among the ICA algorithms that produce asymmetrically distributed outputs with a heavy tail, ours is most closely related to non-negative ICA [40], although in applications we found non-negative ICA to suffer from the problem that positive synaptic currents are completely unconstrained, which precludes interpretations of their magnitudes. A non-negative sparse-coding algorithm with a cost function similar to ours has been described [41], but imposes some tighter restrictions on the mixing matrix 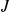 (the inverse of 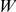) and has the undesirable property that outputs 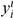 cannot be computed in a single forward pass but require an iterative optimization procedure. Last but not least, our algorithm is different from nonnegative matrix factorization (NMF) [42], because NMF does not allow for STRFs with inhibitory subfields.

### Song selective neurons in the auditory forebrain

In our model, the prominence of BOS in the training set and the shape of our cost function conjunctively forced neurons to display minimal suprathreshold synaptic currents to BOS (on average). These minimal responses explain why neurons preferred CON over BOS at moderately low firing thresholds, very much like field-L neurons do. At higher thresholds, neurons responded to specific BOS features more than to other features. In the high-threshold regime, model neurons were BOS selective, and during BOS presentation they fired sparsely and were hyperpolarized on average, all very much like HVC mirror neurons that project to Area X (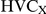 neurons): 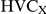 neurons are hyperpolarized by playback of the BOS and produce a high frequency burst in response to a very specific song feature [11], [22]. However, at high thresholds, our model network did no longer function as a universal encoder of the auditory environment. Although BOS reconstructions worsened very gracefully with increasing threshold, the neural representation of non-preferred stimuli degraded rapidly. This observation recommends the high-threshold regime only for specialized auditory areas such as HVC and the low-threshold regime for lower auditory areas such as field L that respond to a large variety of sounds.

Can selectivity for the TUT also be seen as a corollary of an efficient coding hypothesis? HVC neurons in juveniles tend to prefer TUT over most other stimuli including the BOS [21], whereas in adults this selectivity reverses such that HVC neurons tend to prefer BOS over TUT [21], [43]. Taken together, these data could be reproduced by our high-threshold model of HVC if TUT originally were the more prominent stimulus than BOS, but then BOS takes over as the most prominent stimulus.

Our model may explain response properties in many higher auditory brain areas of songbirds including CM: medial CM responses are shaped by auditory memories and cells typically respond more to familiar than to unfamiliar songs [44], in analogy to BOS preference seen in HVC. Interestingly, selective CM cells have lower spontaneous firing rates than non-selective cells, in agreement with the high-threshold regime of our model.

### Smart noise reduction

From an engineering perspective, one computational benefit of the sparseness transformation in our model is smart noise reduction. Using a high firing threshold during reconstruction, sounds to which cells have not been exposed during training can be effectively filtered out. More interestingly, by omitting certain neurons during reconstruction (e.g., by setting their firing thresholds to infinity), undesirable signals encountered in the training set can be conveniently suppressed. For example, by excluding the five neurons that encoded high-frequency noise (e.g. Neuron 106), BOS could be efficiently cleaned from that noise (see Audio S1 and S2). Of course the brain may make use of such smart noise reduction without ever explicitly having to reconstruct the original input; for example, feature-based attentional inputs may selectively suppress the firing in some neurons to constrain downstream processing to only relevant sensory features. Although such selective suppression has not been found yet in songbirds, birds may possess attentional selection mechanisms because they can detect subtle acoustic features and adapt their songs when negatively reinforced [27].

### Song feature analysis

Our algorithm offers a powerful method for bioacoustic signal analysis. The diversity of STRFs in our model is well matched with the behavioral richness of birdsong. Stereotyped syllables can be readily detected because they are represented essentially by a single STRF, whereas more variable syllables such as harmonic stacks may be associated with multiple STRFs as in Figure 4. Hence, the number of STRFs allocated to a particular syllable or sub-syllable may reflect its variability. The level of song analysis (detailed vs coarse) can be controlled by the number of neurons in the network. We can imagine uses of our algorithm for detecting particular song variants (such as high-pitched versions of harmonic stacks), or for identifying similar notes within different syllables, etc. Last but not least, because outputs of the network are more independent than its inputs, our algorithm may be well suited for applications of blind source separation.

### STRFs and their relation to spike responses

Our study is not the first to examine sparse coding of birdsong. Greene et al. applied a popular sparse coding algorithm to compute optimal linear kernels on large numbers of birdsong spectrograms [45]. They evaluated model output in terms of receptive field shapes and found that a stronger sparseness prior during training led to stronger resemblance of model STRFs with STRFs in field L. The STRFs in our similar model also qualitatively resembled STRFs in field L. Without density prior, model STRFs were denser in spectral and temporal modulations than field L STRFs. However, we showed there is no principled discrepancy because by using a suitable density prior, we were able to modulate the STRF density almost arbitrarily (Figure 2B), implying that our model is amenable to fitting a large variety of experimental STRFs. Most importantly, our work suggests that neural firing may constitute a better model read out than receptive fields, because neural firing takes nonlinearities into account (such as the firing threshold), whereas receptive fields are linear and often poor descriptions of spike data. For example, we found that as a function of the firing threshold, our model, despite its fixed underlying STRFs, was able to reproduce qualitatively different responses as seen in field L and HVC. Hence, a simple STRF may be far from ideal as a characterization of neural firing, because it may be associated with a diverse range of response behaviors.

### Applicability to other sensory modalities

Our findings may have relevance for the encoding of sensory modalities other than audition, including olfaction. In mammals, strong odorant-selective responses arise immediately downstream of primary sensory inputs [46]. Though the neural mechanisms of this selectivity remain to be studied, in insects the mechanisms giving rise to sparse odor representations have been well characterized. The sparse odor representation in Kenyon cells arises from synchronized excitatory inputs mediated by densely firing projection neurons in the antennal lobe and by nonspecific inhibitory inputs from lateral horn interneurons that in essence set a high firing threshold to Kenyon cells [47], [48]. The Kenyon cell's supralinear summation of EPSPs [49] represents a simple biophysical mechanism for achieving a long tail in the distribution of positive synaptic currents, a key element of our model. And, the control of firing threshold in Kenyon cells by global inhibitory input is well suited to endow these cells in principle with the ability to change response selectivity, for example if required by external circumstances.

### Function of selectivity reversal

Our model can reproduce the selectivity reversal seen in 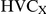 neurons [11]. We predict this to be a widespread phenomenon. We predict that sparsely firing neurons, when they are depolarized by constant current injections to fire densely, will display reduced or negative selectivity for their normally preferred stimulus. Similarly, we predict that densely firing neurons should lose or also reverse their selectivity while being hyperpolarized by constant current injection. These predictions apply to the mean and median selectivities in a large population (not to each individual cell) and should be relatively simple to verify using intracellular recordings. Note that these two predictions are surprising for neurons with monotonic frequency-current (F-I) curves 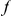 as in our model. We can speculate about the function of such selectivity reversal, if indeed widespread. If it were to be found in other animals and brain areas and were under volitional control, it could be used to attentionally screen the sensory environment for highly familiar stimuli (high threshold case), or to tune in on all kinds of stimuli with preference for unfamiliar ones (low threshold case).

Regarding shifts in excitatory/inhibitory balance, our model predicts that the effect on response selectivity depends on how balance shifts affect firing rates. For example, if increased inhibition leads to decreased firing rates, our model predicts increased response selectivity (for the BOS or an equivalent stimulus). In general, manipulations of excitation or inhibition within a network can lead to highly non-trivial reactions, e.g. disruption of local inhibition onto a cell can lead to lower baseline firing rate and to significant changes in firing patterns. For example, in Rosen and Mooney [50], decreased G-protein coupled inhibition led to decreased baseline firing, which is counterintuitive and may be caused by nonlinear priming effects. Our model is not able to explain such behavior, as it would need to include a more complex neuronal model including synaptic feedback. Nevertheless, because our theory applies in the direction of firing rate changes, our model predicts increased selectivity for the BOS when removal of inhibition decreases firing rates, which is what has been observed [50].

As a model of HVC responses, our findings suggests that vocal-auditory mirrored activity in HVC has a sensory origin (activity in 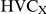 neurons is mirrored in that auditory-evoked and singing-related responses in these cells are nearly identical [22]). In particular, our interpretation is that initially, HVC responses are shaped by the TUT; thereafter, HVC responses and their selectivity are further shaped by auditory feedback elicited by the BOS [21], which during early sensorimotor song development is generated by a motor pathway that excludes HVC [51]. During this developmental phase, a network forms among HVC neurons and ultimately produces adult song. A sensory origin of the HVC network would imply that motor responses in HVC neurons learn to mirror sensory responses, not vice versa (HVC neurons learn to use auditory-feedback-elicited responses as future motor outputs, rather than their learning to map auditory feedback onto the HVC neurons that were involved in generating the feedback). In other words, when mirror neurons fire during motor behavior, they do so mainly because they have developed selectivity to the stimulus preceding their firing. More specifically, mirrored activity in HVC neurons could derive from essentially one assumption: that the local HVC network tries to maximize the drive of cells at the moments at which these fire, initially driven by sensory input. Accordingly, HVC synapses would allow for cells to drive each other at time lags at which their preferred TUT/BOS auditory features occur. Such specific function could arise for example by virtue of some spike-time dependent synaptic plasticity mechanisms [52]–[55].

In conclusion, the architecture we have described shows that efficient coding constraints can explain the diversity of response specificity in higher sensory areas. Sparse/selective and dense/antiselective responses are at opposite extremes of the same efficient coding principle. It is possible that this link between response specificity and firing sparseness holds true also in other neural systems such as the neocortex. And, by extrapolation, our work shows that efficient coding constraints may guide the formation of sensory pathways all the way up to premotor areas, by which our work can shorten the gap between our understanding of pure sensory and pure motor codes.

## Methods

### Cochlear input

The cochlear input to the network at time 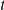 was formed by the most recent 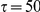 ms window of the (mean-subtracted) log-power sound spectrogram 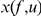, where time 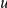 ranged from 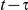 to 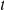, and the frequency 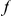 from 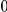 Hz to 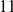 kHz. To form these spectrograms, the sound was sampled at 22 kHz, multiplied with a Hanning window of 256 or 128 samples, and Fourier transformed, where in each frequency band we subtracted the mean spectral log-power. The frequency resolution of spectrograms was 86 or 172 Hz, respectively. The temporal offset 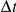 between subsequent cochlear inputs was 16 or 32 samples, corresponding to 0.7 and 1.5 ms, respectively. The dimension 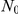 of the cochlear input was 8192 for high-resolution spectrograms (86 Hz, 0.7 ms, Figures 2B and 5 to [Fig pone-0025506-g006] [Fig pone-0025506-g007] [Fig pone-0025506-g008] [Fig pone-0025506-g009] [Fig pone-0025506-g010] [Fig pone-0025506-g011] [Fig pone-0025506-g012] 13) and 2048 for low-resolution spectrograms (172 Hz, 1.5 ms, Figures 2A and 3). For Figure 4 the spectrum ranged from 0 kHz to 8 kHz at a resolution of 62.5 Hz, and a temporal resolution of 4 ms was used.

### Network model

The synaptic current 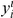 to neuron 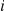 (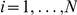, where 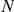 varied from 100 to 800) was formed by convolving the cochlear input 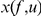 in time with synaptic weights 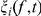, and by summing over frequency bands:
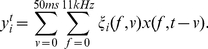
(1)We interpret the synaptic weights 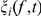 as the STRF of cell 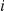 [56]. To simplify our notation, we vectorize the cochlear input 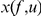 as a column vector 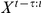 and the synaptic weights 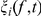 as a row vector 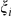, allowing us to rewrite Equation 1 as the scalar product
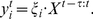
(2)


We considered two separate models for transforming synaptic currents into firing rates. In our threshold-linear or analog model, the firing rate 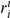 of cell 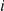 at time 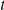 was proportional to the total synaptic current above a firing threshold 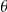,
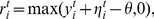
(3)where 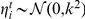 represents a source of independent Gaussian white noise of mean zero and standard deviation 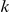. This noise modeled fluctuating firing rates when 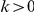. Note that we also simulated a second binary model, in which neurons fired at a constant rate when the total synaptic current exceeded 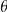, 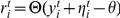, where 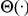 is the Heaviside function, 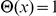 if 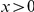 and 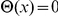 otherwise.

Neural firing in our model is set by a separating hyperplane of the input space 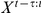. Note that such a linear thresholding scheme might not be optimal for non-Gaussian stimulus ensembles [57]. We may study extensions from planar to curved decision boundaries in future work, provided suitable mathematical tools can be devised.

### Networks of two layers

We also analyzed hierarchical networks with two layers, with the goal to evaluate BOS selectivity in the second layer as a model of HVC.

The input 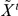 to the second layer was formed by a 50 ms window of (mean-subtracted) first-layer firing rates 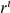, down-sampled from 0.7 ms to 2.8 ms (by averaging). Neuron 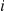 in the second layer (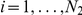) had a non-zero firing rate (threshold-linear or binary) at time 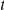 when its total synaptic current 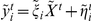 exceeded a threshold 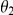 (with 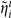 representing a source of Gaussian white noise of mean zero and standard deviation 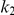). The total number of second-layer neurons 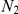 was set to 800. Unlike in the first layer, the second-layer synaptic weights 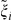 cannot be interpreted as receptive field due to the dependence of second-layer responses on first-layer synaptic weights. To train the second-layer weights, we first whitened first-layer responses and then picked random 50-ms sets thereof (each with a new random noise) to iteratively update the sparseness transformation 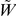. Hence, the training data for the second layer depended on the first-layer firing threshold 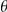. For training of the second layer, we observed good convergence behavior when 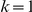 and 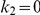 (though results did not qualitatively depend on noise amplitudes during learning).

### Synaptic weights and the cost function

The 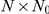 synaptic weight matrix 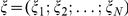 was given by matrix multiplication of a projection 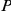, whitening the cochlear input, followed by a sparseness transformation 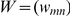:
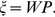
(4)


The projection 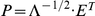 was determined by principal-component analysis, where 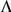 is the diagonal matrix of the 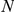 largest eigenvalues of the covariance matrix 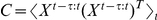 of the cochlear, 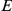 is the 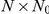 matrix of corresponding orthonormal eigenvectors, and 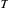 denotes the transpose.

For a given projection 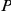, the sparseness transformation 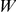 was computed iteratively by minimizing the following cost function for synaptic currents 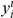:
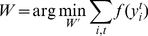
(5)where
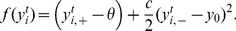
(6)In this last equation, 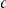 is the trade-off between the two summands, 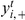 are suprathreshold synaptic currents (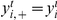 for 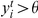 and 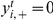 otherwise), and 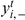 are subthreshold synaptic currents (
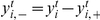
).

This cost function contains a linear cost for suprathreshold currents (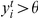) and a square cost for subthreshold synaptic currents (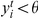). The function has two minima of zero cost at 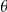 and at 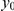. 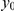 is a parameter that sets the current which elicits the minimum subthreshold cost. Changing 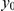 has no qualitative influence on our findings; in practice 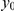 can be set to zero both during learning and evaluation of the network (thus considerably simplifying the learning procedure described below). However, to achieve minimal reconstruction error as we will show, in some simulations we set 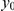 to the expected subthreshold synaptic current, defined by 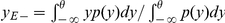, with 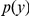 being the probability density function of synaptic currents elicited by the training set. Hence, 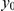 should be regarded as a useful parameter if close to optimal reconstructions are desirable, but otherwise this parameter can be confidently neglected.

For most simulations we set 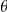 to zero during learning, implying that suprathreshold currents are depolarizing (positive), whereas subthreshold currents are hyperpolarizing (negative), Figure 1C. The factor 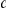 was normally set to one, but the simulations showed a high robustness even when increasing 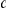 by two orders of magnitude.

The function 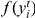 in Equation 6 has a discontinuity in 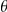, which hinders gradient-descent algorithms. In practice, we smoothed 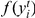 locally at the threshold 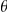 using a sigmoidal function 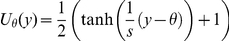 as follows:
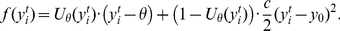
(7)The variable 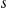 is a smoothing constant that should be in the range of average sampling density in the neighborhood of the threshold (in our simulations we chose 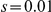 for 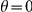 and 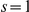 for 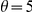).

To avoid the trivial optimum 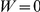 in Equation 5, we set the following restriction to the (left) inverse matrix 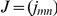


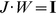
(8)

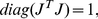
(9)where 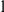 is a vector of ones and 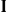 the unity matrix. This constraint is motivated by functional/theoretical consideration and could potentially be accomplished by a host of complex homeostatic mechanisms, though such an analysis would go beyond the limits of this work. In different sets of simulations we also examined the weaker (volume-preserving) restriction 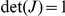 and the stronger (orthonormal) restriction 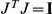, where 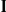 is the identity matrix. For the entire range of thresholds 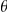 tested, the distributions of BOS-REV and BOS-CON selectivities were almost unchanged under these two restrictions, demonstrating robustness of our results with respect to the synaptic weight constraint. Also, similarity of our findings with findings obtained when using the Bell-Sejowski algorithm demonstrates high robustness. Note that all presented simulation used a squared projection matrix 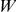 were 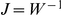 is the inverse.

Geometrically, the cost function penalizes subthreshold outliers more than suprathreshold ones, because the square Euclidean norm of a large vector is larger than its Manhattan norm. Furthermore, the cost of mapping an outlier to a suprathreshold synaptic current in just a single neuron is smaller than the cost of distributing the outlier among multiple neurons. This can be best seen for the special case of sparseness transformations that are rotations (i.e. orthonormal, satisfying 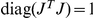), because the Manhattan norm of a vector of fixed Euclidean norm is minimized when it is parallel to one of the coordinate axes. Thus, the cost function tries to produce highly selective neurons that are strongly excited by only a very small set of inputs.

To have control over the density of STRFs (sharply tuned versus broadly tuned neurons) we run a few simulations with an additional term in the cost function, corresponding to a linear cost on absolute synaptic weights,
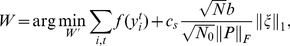
(10)where 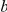 denotes the batch size (number of cochlear input samples per weight update), 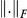 the elementwise Frobenius-norm, and 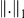 an elementwise 1-norm and 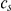 is the relative weight of the new term. In all our simulation results reported in the Results Section and the figures, we applied 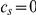, except results shown in Figures 2B and 5, where we used 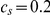. In summary, for 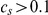 STRFs were of low density (Figure 2B) in closer resemblance to experimental data (with this choice of 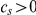 our findings about selectivity reversal still applied).

### STRF estimation using reverse correlation

We examined the question whether estimated STRFs using reverse correlation depend strongly on the firing threshold and whether they resemble the STRFs encoded in the synaptic weights. For various thresholds 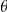 we calculated the firing rates 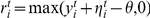 in response to birdsong stimuli and estimated the reverse-correlation STRFs 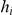 in terms of
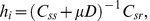
(11)where 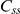 is the stimulus autocorrelation matrix, 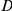 the diagonal of the autocorrelation matrix, 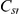 the crosscorrelation between the stimulus and the response, and 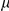 is a small factor used to regularize the inverse. Using the estimated STRFs we predicted the firing rates 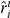 to novel stimuli. We compared predicted firing rates to firing rates elicited by the actual STRFs using the correlation coefficient 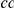.

### Minimal decoding error

The first term of the cost function in Equation 6 imposes a linear cost on suprathreshold synaptic currents. Because suprathreshold synaptic currents are equivalent to instantaneous firing rates at zero noise, the first term enforces firing sparseness across the population (for the training threshold). The second term imposes a quadratic cost on subthreshold synaptic currents, which is equivalent to minimizing an error bound on decoded cochlear inputs. To see this, consider the following estimate 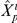 of whitened cochlear inputs 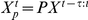 from suprathreshold synaptic currents at time 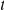:
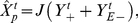
(12)where 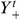 is a time-dependent vector of suprathreshold currents 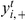 and the vector 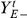 is the vector with the expected subthreshold currents: 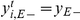 for 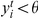 and 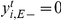 else.

The decoding error associated with the decoding 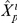 is given by the mean square Euclidean norm between 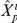 and 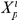. This error is related to the distribution of subthreshold currents as follows:
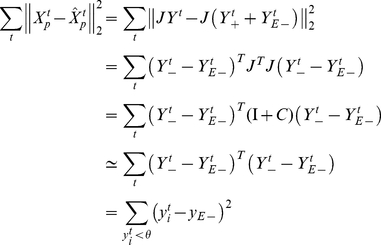
(13)where in the first line we have used that 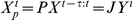, and in the second line 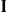 represents the identity matrix and 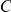 a matrix with zeros on the diagonal. The vector 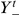 is the vector of the real subthreshold currents 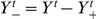. The approximation in the second line is based on the assumption of equally distributed and mutually independent subthreshold currents: 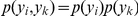. Note that the approximation in Equation 13 is exact for rotations (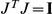), whereas for the constraint 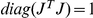, we found the approximation to be within 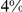 of the reconstruction error for 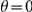, and to be even closer for higher 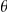.

The key insight is that for the training threshold 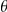, the approximated reconstruction error is proportional to the subthreshold term of our cost function if we choose 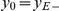 in Equations 5 and 6. Hence, by minimizing the subthreshold term in our cost function, we minimize the approximation of the decoding error. The final term in Equation 13 shows that the reconstruction error is small when the subthreshold currents are rare (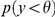 is small) and their variance is small (
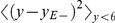
 is small).

Note that the decoding scheme defined in Equation 12 may not be globally optimal, but was motivated by our assumption that only suprathreshold events carry meaningful information. Also, a benefit of this decoding scheme is its asymptotic robustness (for a threshold of minus infinity the decoding error vanishes).

Our algorithm minimizes an approximation of the reconstruction error, but not the reconstruction error itself. Exploratively, we have adapted the algorithm to directly minimize the reconstruction error (defined in Equations 13) for a given threshold 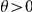. The resulting reconstruction error for BOS at the given threshold was only marginally better than with our sparse-coding algorithm. Synaptic current distributions were nearly bimodal with a first peak at zero and a second peak slightly above 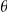; interestingly, reconstructions became worse than in Figure 9E when performed using a different threshold than used during learning.

### Reconstructed Spectrograms

From the decoded whitened inputs 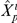, we reconstructed the spectrograms (Figure 9) using the pseudoinverse 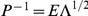. The element 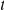 of the reconstructed spectrogram was defined by
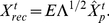
(14)From these elements, we computed the fully reconstructed spectrograms by averaging over all overlapping regions in the sequence 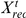, 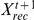, 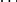 (average over 64 time windows).

### Algorithm for cost minimization

The constraint in Equation 8 was naturally enforced by our choice of parameterization of the inverse:
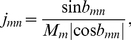
(15)where 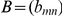 is an arbitrary parameter matrix and 
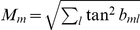
 is a row-wise normalization constant. We minimized the cost function 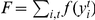 iteratively using a line search along the direction 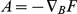 of steepest descent along the cost surface, which was given by:
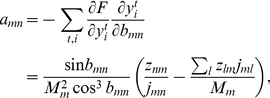
(16)where
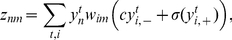
(17)for the simplified cost function (Equation 6) and for 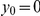. 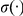 denotes the signum function. However, for the general, smoothed cost function (Equation 7) the calculation of 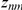 gets more complex:
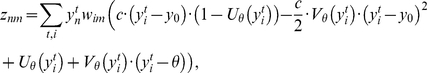
(18)where 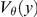 is the derivative of the sigmoid function 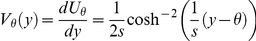.

Starting with the identity matrix 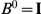, we updated the 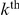 parameter matrix 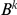 according to
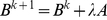
(19)using an optimized step size 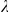 determined through a line search algorithm:
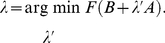
(20)After each update of 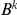, we updated the inverse 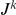 according to Equation 15 and the sparseness transformation 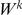 by inversion of 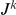. In simulation in which the minimum subthreshold cost 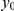 was variable, we changed 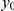 at each update to the expected subthreshold current, 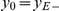. During evaluation of the network we left 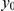 untouched.

Note that we also formulated an overcomplete version of our algorithm, in which there are more neurons than principal components (the matrix 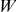 has more rows than columns). In this case the resulting synaptic current distribution had an even heavier tail on the positive side, but results were qualitatively unchanged.

### Batch training

Our training data consisted of 46 sound files of average duration 1.7 s. Thirty-four of these files contained different renditions of a zebra finch song (BOS), twelve files contained conspecific songs (CON) from two independently raised adult zebra finches in our colony (six files each). Our results did not depend sensitively on these file numbers and which song was chosen as the BOS, as long as there was an overall predominance of BOS.

To train the synaptic weight matrices, we used a randomized batch learning scheme. First we computed the whitened cochlear inputs based on the matrix 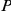 derived from all 46 sound files. To train the sparseness transformation, we chose a batch size of a few thousand 50-ms windows. Each window in this set was drawn randomly from one of the 46 files and at a random time within the file. From this set of whitened inputs we computed the gradient matrix 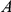 (Equation 16), performed the line search (Equation 20), and updated 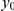 if necessary. This procedure was then repeated with different sets of random windows. Typically, the number of updates required to reach convergence was within two to ten times the number of neurons 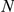.

In separate simulations we also included in the training set various numbers of files with cage noises such as wing flaps, subsongs and other natural sounds; results did not qualitatively change with the inclusion of these files.

### Response selectivity

We measured the selectivity of neural responses using the psychophysical 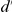 measure [32]. This measure discriminates between two different stimuli 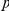 and 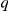 based on the average firing rates 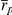 and 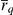 they elicit in individual neurons (averages are formed over stimulus presentations and over their durations):
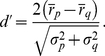
(21)The terms 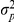 and 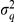 in the denominator are the variances of mean firing rates across repeated stimulus presentations (this variance was nonzero in our simulations due to the additive noise in Equation 3). A positive 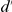 value implies selectivity for 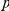, whereas a negative value implies selectivity for 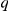. Selectivity results did not depend qualitatively on the noise amplitude (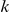 from 0.05 to 5). The effect of increasing the noise amplitude 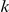 was to bring 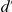 values closer to zero, but without changing their signs. In Figure 11, BOS-CON selectivity was evaluated on 3 different CONs that were not part of the training set.

To evaluate the 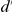 selectivity in the Bell-Sejnowski architecture, we defined the firing rate of neuron 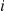 by the symmetrically rectified expression 
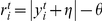
 for 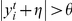, and 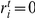 otherwise. All programming was done in Matlab (Mathworks Inc).

## Supporting Information

Audio S1 **Original Recording of a zebra finch song.**
(WAV)

Audio S2 **Reconstruction of the song in Audio S1 and after smart noise suppression.**
(WAV)
